# Maximum likelihood estimation of biophysical parameters of synaptic receptors from macroscopic currents

**DOI:** 10.3389/fncel.2014.00303

**Published:** 2014-10-02

**Authors:** Andrey Stepanyuk, Anya Borisyuk, Pavel Belan

**Affiliations:** ^1^Laboratory of Molecular Biophysics, Bogomoletz Institute of PhysiologyKiev, Ukraine; ^2^State Key Laboratory of Molecular and Cellular Biology, Bogomoletz Institute of PhysiologyKiev, Ukraine

**Keywords:** unitary current, synaptic currents, peak-scaled non-stationary fluctuation analysis, maximum likelihood, semiseparable matrix, kinetic model, Markov chain Monte Carlo

## Abstract

Dendritic integration and neuronal firing patterns strongly depend on biophysical properties of synaptic ligand-gated channels. However, precise estimation of biophysical parameters of these channels in their intrinsic environment is complicated and still unresolved problem. Here we describe a novel method based on a maximum likelihood approach that allows to estimate not only the unitary current of synaptic receptor channels but also their multiple conductance levels, kinetic constants, the number of receptors bound with a neurotransmitter, and the peak open probability from experimentally feasible number of postsynaptic currents. The new method also improves the accuracy of evaluation of unitary current as compared to the peak-scaled non-stationary fluctuation analysis, leading to a possibility to precisely estimate this important parameter from a few postsynaptic currents recorded in steady-state conditions. Estimation of unitary current with this method is robust even if postsynaptic currents are generated by receptors having different kinetic parameters, the case when peak-scaled non-stationary fluctuation analysis is not applicable. Thus, with the new method, routinely recorded postsynaptic currents could be used to study the properties of synaptic receptors in their native biochemical environment.

## Introduction

Intrinsic biophysical properties of synaptic receptor channels are important for determining of both efficacy of synaptic transmission and activation of dendritic voltage-gated channels underlying active properties of dendrites. For example, synaptic NMDA receptors directly contribute to non-linear depolarizing drive in dendrites and control dendritic firing patterns and local dendritic Ca^2+^ concentration transients (Major et al., 2013). Changes in the postsynaptic receptor number, unitary conductance, and kinetics may affect dendritic integration (Magee, 2000) and lead to alteration in synaptic strength and memory function (Li and Tsien, 2009) in normal (Benke et al., 1998) and pathological states (Kittler et al., 2004). Thus, precise estimation of these parameters is important for a better understanding of synaptic transmission and dendritic excitability.

However, postsynaptic receptors in their native environment are hardly accessible experimentally, and this limitation has rendered their biophysical properties notoriously difficult to study. In order to cope with this problem, postsynaptic receptors are heterologously expressed and studied using single channel recording in small membrane patches by means of fast application of respective neurotransmitters. At the same time it has been claimed using proteomic approaches that postsynaptic receptors can interact with dozens of intracellular proteins (Husi et al., 2000) that results in modulation of their functioning. Besides, many extracellular factors such as, e.g., ions, certainly affect synaptic receptor function (Paoletti et al., 1997; Low et al., 2000). Altogether it makes it almost impossible to directly apply receptor biophysical parameters obtained in a heterologous system to the analysis of postsynaptic receptors under physiological conditions.

The peak-scaled non-stationary fluctuation analysis (PS NSFA) (Traynelis et al., 1993) is a most commonly used indirect method by which unitary current of synaptic receptors can be extracted from the macroscopic synaptic currents. This continuation of the conventional non-stationary noise analysis (Sigworth, 1980) overcomes impact of the quantal variability of postsynaptic currents on the accuracy of unitary current estimation by scaling the mean postsynaptic current waveform to the peak amplitude of each individual postsynaptic current. The two waveforms are then subtracted to isolate fluctuations arising from the synaptic channel gating. However, by using PS NSFA information about the total number of synaptic receptors bound or exposed to a neurotransmitter is lost and only the average number of receptors open at the peak of the postsynaptic current can be estimated (Traynelis et al., 1993; Silver et al., 1996). Activation, inactivation, and desensitization, key features of synaptic receptor behavior, which are determined by receptor kinetic parameters, also could not be evaluated by PS NSFA. Although many attempts have been performed to estimate the kinetic constants of ion channels from fluctuations of postsynaptic macroscopic currents (Neher and Stevens, 1977; Traynelis and Jaramillo, 1998; Milescu et al., 2005; Moffatt, 2007) all of them do not get over the quantal variability of postsynaptic currents or could not be easily applied to the analysis of these currents because of restricted accuracy and efficiency.

By overcoming computational complexity that emerges due to quantal variability of postsynaptic currents, a maximum likelihood non-stationary fluctuation analysis (ML NSFA) suggested in this work makes it possible to estimate unitary currents, number of channels bound with a neurotransmitter, peak open probability, and some kinetic constants for synaptic channels in their native biochemical environment from the experimentally feasible number of macroscopic postsynaptic currents.

## Materials and methods

### Kinetic model

In this work we consider simulated macroscopic synaptic currents generated by a varying number of synaptic receptor channels. The channels are assumed to be independent and identical. We assume that the synaptic channel gating is a Markov process and *p_ij_* is the probability of channel transition from state *j* to state *i* at time Δ*t*. The rate matrix is an N_s_ × N_s_ matrix **Q**: $q_{ij}=\underset{\Delta t\to 0}{\text{lim}}\frac{p_{ij}}{\Delta t},$ N_s_ is the number of states of the synaptic channel model. Each element of the matrix **Q** gives the rate constant of transition *j* → *i* if the transition is allowed by the model and otherwise *q_ij_* = 0. The diagonal elements, *q_ii_*, are set to − $\sum_{j}q_{ij},$ so the sum over each column is zero. Synaptic release of neurotransmitter is modeled as a step pulse of its concentration in the synaptic cleft, which leads to instantaneous change of concentration-dependent transition probabilities, *p_ij_*.

We assume that the kinetic matrix topology (i.e., a set of allowed transitions) and the number of conducting states are known. The required model parameters were arranged into the parameter vector θ = [*q, i*_ch_, N_ch_] and they were: rate constants, $q_{ij}=\underset{\Delta t\to 0}{\lim}\frac{p_{ij}}{\Delta t},i\neq j,$ unitary currents, *i_ch_*, and the number of postsynaptic receptors bound with a neurotransmitter right after the concentration transient, N_ch_.

### The log-likelihood function

The likelihood function, L_θ_, that is to be maximized by ML NSFA in order to find the most likely set of parameters is defined as the conditional probability to observe *N* macroscopic current traces ***c****_i_, i* = 1 : *N*, sampled at time points *t* = [1… *N_T_*] given the model parameters θ (Colquhoun and Hawkes, 1977; Celentano and Hawkes, 2004; Milescu et al., 2005; Stepanyuk et al., 2011):

(1) $$\begin{array}{l} {\text{L}}_{\theta }\equiv P(c|\theta )\underset{{\text{N}}_{\text{ch}}\to \infty }{\to }\frac{1}{{\left(2\pi \right)}^{{\text{NN}}_{\text{T}}/2}\prod_{i=1}^{N}{\left|c_{\text{m}1}{\text{N}}_{\text{chi}}\right|}^{1/2}}\\ \text{ exp}\left\{-\frac{1}{2}\sum_{i=1}^{N}{\left(c_{i}-\mu {\text{N}}_{\text{chi}}\right)}^{\text{T}}\frac{c_{\text{m}1}^{-1}}{{\text{N}}_{\text{chi}}}\left(c_{i}-\mu {\text{N}}_{\text{chi}}\right)\right\} \end{array}$$

Here *N* is the number of synaptic macroscopic current traces ***c****_i_* (sample size) and N_T_ is the number of points in each trace; N_chi_ is a number of channels exposed to neurotransmitter in each current ***c****_i_*; μ, an N_T_ × 1 vector and **c**_m1_, an N_T_ × N_T_ matrix with elements {**c**_m1_}*_t,t′_*, and denote mean and covariance of single channel current, respectively, and they both are the functions of **θ**. **c**_m1_ is related to the covariance matrix, **c**_m_, of a macroscopic synaptic current ***c**_i_* by the following expression: **c**_m_ = **c**_m1_ N_chi_. Mean and covariance follow equations (Colquhoun and Hawkes, 1977)

(2) $$\begin{array}{l} \mu =i^{\text{T}}e^{Q\text{t}}p\left(0\right)\\ {\left\{c_{\text{m}1}\right\}}_{t,t^{\prime }}=\left(i^{\text{T}}{\text{e}}^{Q\text{t}}p\left(0\right){\text{e}}^{Q\left({\text{t}}^{\prime }-\text{t}\right)}i\text{​}-\text{​}\left(i^{\text{T}}{\text{e}}^{Q{\text{t}}^{\prime }}p\text{​}\left(0\right)\right)\text{​}\left(i^{\text{T}}{\text{e}}^{Q\text{t}}p\left(0\right)\right)\right) \end{array}$$

Here **Q** is a rate matrix (Colquhoun and Hawkes, 1977; Celentano and Hawkes, 2004) and ***p*** (0) is an initial state vector. The elements of ***p*** (0) can be calculated as the equilibrium probabilities determined by the initial experimental conditions, which are assumed to last for sufficiently long time *T* to allow the channels reach equilibrium

(3) $$p\left(0\right)=\prod_{j}{\text{e}}^{Q_{j}\Delta t_{j}}p(-T)$$

It is generally accepted to maximize the logarithm of the likelihood function logL_θ_ instead of the likelihood function L_θ_ itself. Therefore, our objective was to find the most likely model parameter set, **θ**_*ML*_, i.e., the parameter set that maximized the log-likelihood

(4) $$\theta_{\text{ML}}=\underset{\theta }{\text{argmax}}\left({\text{logL}}_{\theta }\right)$$

The log-likelihood function logL_θ_ can be efficiently estimated using the fact that **c**_*m*1_ has a specific structure of semiseparable matrix (DeWilde and van der Veen, 1998; Stepanyuk et al., 2011).

### Efficient estimation of the log-likelihood function for synaptic currents with noise

Efficient log-likelihood estimation used in this article is based on our previously described method (Stepanyuk et al., 2011). Briefly, the method was based on the fact that the covariance matrix **c**_m1_, has a specific structure of semiseparable matrix, namely **c**_m1_ can be represented as Stepanyuk et al. (2011).

(5) $$\begin{array}{l} {\left\{c_{\text{m}1}\right\}}_{ij}=\sum_{k=1}^{N_{S}+1}{\text{A}}_{ik}{\text{B}}_{kj},\text{i}\geq \text{j}\\ {\left\{c_{\text{m}1}\right\}}_{ji}={\left\{c_{\text{m}1}\right\}}_{ij},\text{i}\geq \text{j} \end{array}$$

where

(6) $$\begin{array}{l} {\text{A}}_{\text{ik}}={\text{e}}^{\lambda_{\text{k}}{\text{t}}_{\text{i}}}\sum_{\text{o}=1}^{{\text{N}}_{\text{O}}}{\text{i}}_{\text{o}}\text{U}\left(\text{o},\text{k}\right),\text{1}\leq \text{k}\leq {\text{N}}_{\text{S}}\\ {\text{A}}_{\text{ik}}=\mu_{{\text{t}}_{\text{i}}},\text{k}={\text{N}}_{\text{S}}+\text{1} \end{array}$$

and

(7) $$\begin{array}{l} {\text{B}}_{\text{kj}}={\text{e}}^{\lambda_{\text{k}}\left({\text{T}}_{\text{s}(\text{j})}-{\text{t}}_{\text{j}}\right)}\left(\sum_{{\text{o}}^{\prime }=1}^{{\text{N}}_{\text{O}}}{\text{U}}^{-\text{1}}\left(\text{k},{\text{o}}^{\prime }\right){\text{p}}_{\text{o}}^{\prime }\left({\text{t}}_{\text{j}}\right){\text{i}}_{\text{o}}^{\prime }\right),\text{1}\leq \text{k}\leq {\text{N}}_{\text{S}}\\ {\text{B}}_{\text{kj}}=-\mu_{{\text{t}}_{\text{j}}},\text{k}={\text{N}}_{\text{S}}+\text{1} \end{array}$$

where **U**: e^Qt^ = **U**e^**Dt**^**U**^−1^ is N_s_ × N_s_ matrix of the eigenvectors of **Q**, and **D** is a diagonal form of **Q** provided all the eigenvalues of **Q**, λ, are different; *N*_o_ is a number of open states in the channel model. Efficient linear algebra algorithms for semiseparable matrices (Vandebril et al., 2007; Eidelman and Gohberg, 2008) allowed us to compute the log-likelihood and provided almost linear scaling of its computational cost with the number of states in a kinetic model for the case of sufficiently large number of currents, ensuring fast, and accurate estimation of model parameters. The number of synaptic channels exposed to neurotransmitter was assumed to be the same for all currents. However, in the case of synaptic currents this number could vary between trials due to quantal variability. As a result, logL_θ_ must be estimated separately for each current, and then summed up, thus increasing the number of operations in *N* times at least. However, calculations could be substantially simplified if the majority of receptors, which will participate in the current, are found in one particular state immediately after the neurotransmitter concentration transient, as it is expected for the synaptic receptors. To compute logL_θ_ in this case, let denote noisy macroscopic synaptic current with an N_T_ × 1 vector ***c_i_*** and let denote by ***n_i_*** an N_T_ × 1 vector of noise imposed on the *i*-th current. Then −logL_θ_ of the set of parameters **θ** given macroscopic synaptic currents without noise imposed on them is (we omit here the constant term NN_T_ log (2π)/2)

(8) $$\begin{array}{l} -\log{\text{L}}_{\theta }\left(c-n\right)=\frac{1}{2}\sum_{i=1}^{N}{\left(c_{i}-n_{i}-\mu {\text{N}}_{{\text{ch}}_{\text{i}}}\right)}^{\text{T}}\frac{c_{\text{m}1}^{-1}}{{\text{N}}_{{\text{ch}}_{\text{i}}}}\\ \;\left(c_{i}-n_{i}-\mu {\text{N}}_{\text{chi}}\right)+\frac{1}{2}\sum_{i=1}^{N}\left(\log|c_{\text{m}1}{\text{N}}_{\text{chi}}|\right) \end{array}$$

where μ is an expectation of synaptic current without noise and logL_θ_ (**c** − **n**) denotes the required log-likelihood given the set of macroscopic synaptic currents without noise. Equation (8) can be rewritten as

(9) $$\begin{array}{l} -\log{\text{L}}_{\theta }\left(c-n\right)=-\log{\text{L}}_{\theta }\left(c\right)-\frac{1}{2}\sum_{i=1}^{\text{N}}n_{i}^{\text{T}}c_{\text{m}1}^{-1}n_{i}\frac{1}{{\text{N}}_{{\text{ch}}_{\text{i}}}}\\ \;-\sum_{i=1}^{\text{N}}n_{i}^{\text{T}}\frac{c_{\text{m}1}^{-1}}{{\text{N}}_{{\text{ch}}_{\text{i}}}}\left(c_{i}-n_{i}-\mu {\text{N}}_{{\text{ch}}_{\text{i}}}\right) \end{array}$$

Since the background noise and the macroscopic current are uncorrelated the last term in Equation (9) can be neglected without loss in accuracy given the number of currents, *N*, is large enough. Therefore, we have

(10) $$-\log{\text{L}}_{\theta }\left(c-n\right)=-\log{\text{L}}_{\theta }\left(c\right)-\frac{1}{2}\sum_{i=1}^{\text{N}}n_{i}^{\text{T}}c_{\text{m}1}^{-1}n_{i}\frac{1}{{\text{N}}_{{\text{ch}}_{\text{i}}}}$$

To quickly evaluate the last term in Equation (10), let us approximate $\sum_{i=1}^{\text{N}}\frac{{\left(n_{i}{}^{T}\right)}_{k}{\left(n_{i}\right)}_{j}}{{\text{N}}_{ch_{i}}}\text{by}{\left\{c_{noise}^{}\right\}}_{kj}\text{N}\langle \frac{\text{1}}{{\text{N}}_{\text{ch}}}\rangle .$ Hence,

(11) $$\begin{array}{l} \sum_{i\;=\;1}^{\text{N}}n_{i}^{\text{T}}c_{\text{m}1}^{-1}n_{i}\frac{1}{{\text{N}}_{{\text{ch}}_{\text{i}}}}=\sum_{k\;=\;1}^{{\text{N}}_{\text{T}}}\sum_{j\;=\;1}^{{\text{N}}_{\text{T}}}{\left\{c_{\text{m}1}^{-1}\right\}}_{kj}\sum_{i\;=\;1}^{\text{N}}{\left(n_{i}^{\text{T}}\right)}_{k}{\left(n_{i}\right)}_{j}\frac{1}{{\text{N}}_{{\text{ch}}_{\text{i}}}}\\ \;=\sum_{k=1}^{{\text{N}}_{\text{T}}}\sum_{j=1}^{{\text{N}}_{\text{T}}}{\left\{c_{\text{m}1}^{-1}\right\}}_{kj}{\left\{c_{\text{noise}}\right\}}_{kj}\text{N}\langle \frac{1}{{\text{N}}_{\text{ch}}}\rangle \end{array}$$

Finally, from Equation (11) we obtain

(12) $$\sum_{i=1}^{\text{N}}n_{i}^{T}c_{\text{m1}}^{-1}n_{i}\frac{1}{{\text{N}}_{{\text{ch}}_{\text{i}}}}=\sum \sum \left(c_{\text{m1}}^{-1}{}^{\circ}c_{\text{noise}}\right)\text{N}\langle \frac{1}{{\text{N}}_{\text{ch}}}\rangle$$

where ° denote Hadamard multiplication.

Keeping in mind that $\sum \sum c_{m1}^{-1}{}^{\circ}c_{noise}^{}=\text{tr}\left(c_{m1}^{-1}c_{noise}^{}\right),$ we rewrite Equation (10) for logL_θ_ (**c** − **n**) as

(13) $$-\log{\text{L}}_{\theta }\left(c-n\right)=-\log{\text{L}}_{\theta }\left(c\right)-\frac{1}{2}\text{tr​}\left(c_{\text{m1}}^{-1}c_{\text{noise}}\right)\text{N}\langle \frac{1}{{\text{N}}_{\text{ch}}}\rangle ,$$

where

(14) $$\begin{array}{l} \log{\text{L}}_{\theta }\left(c\right)=-\frac{1}{2}\sum_{i=1}^{N}\{{\left(c_{i}-\mu {\text{N}}_{{\text{ch}}_{\text{i}}}\right)}^{T}\frac{c_{\text{m1}}^{-1}}{{\text{N}}_{{\text{ch}}_{\text{i}}}}\left(c_{i}-\mu {\text{N}}_{{\text{ch}}_{\text{i}}}\right)\\ \;+{\text{N}}_{\text{T}}\log{\text{N}}_{{\text{ch}}_{\text{i}}}\text{​​​​​​​​​​​​}\}-\frac{\text{N}}{2}\log|c_{\text{m}1}| \end{array}$$

is the log-likelihood function of macroscopic synaptic currents with noise.

To quickly evaluate $\text{tr}\left(c_{\text{m}1}^{-1}c_{\text{noise}}\right)$ we note that matrices **c**^−1^_m1_ and **c**_**noise**_ are quasiseparable (as an inverse of semiseparable matrix, Vandebril et al., 2007) and semiseparable matrice, respectively. Semiseparability of noise covariance matrix, **c**_**noise**_, follows from the fact that experimental background noise can be well approximated by a stationary Gaussian process, and the covariance matrix of such process is semiseparable (DeWilde and van der Veen, 1998). Then, the computation of trace of the product of such matrices can be accelerated by representing it as $\text{tr}\left(F\;\cdot \;C\right)=2\text{tr}(H\;\cdot \;B)+\sum_{k=1}^{N_{T}}F_{kk}d_{k},$ where **H** is (N_T_ − 1) × N_S_ matrix, **F** is symmetric N_T_ × N_T_ semiseparable or quasiseparable matrix and **B** is defined by Equation (7) (see also Equations A1.26–A1.35 from Text S1 in Appendix in Stepanyuk et al., 2011).

### Estimation of the number of channels and peak open probability

To estimate the number of channels N_chi_ (see Results for further definition), we re-write Equation (8) for a single macroscopic synaptic current:

(15) $$\begin{array}{l} -\log{\text{L}}_{\theta }\left(c_{i}-n_{i}\right)=\text{​}\frac{1}{2}(\frac{c_{i}^{\text{T}}c_{\text{m}1}^{-1}c_{i}}{{\text{N}}_{\text{chi}}}+\mu^{\text{T}}c_{\text{m}1}^{-1}\mu {\text{N}}_{\text{chi}}\\ \;-2\frac{{\left(c_{i}-n_{i}-\mu {\text{N}}_{\text{chi}}\right)}^{\text{T}}c_{\text{m}1}^{-1}n_{i}}{{\text{N}}_{\text{chi}}}-\frac{n_{i}^{\text{T}}c_{\text{m}1}^{-1}n_{i}}{{\text{N}}_{\text{chi}}}\\ \;-2\mu^{\text{T}}c_{\text{m}1}^{-1}c_{i}\text{​​​​​​​​​​​​​​​​​​​​​​​})+\frac{{\text{N}}_{\text{T}}}{2}\log{\text{N}}_{\text{chi}}+\frac{1}{2}\log\left|c_{\text{m}1}\right| \end{array}$$

In the last expression we neglect the 3-d term, as it was done in Equation (9), and the 5-th and the last terms does not depend on N_chi_ at all. Leaving terms that depend on N_chi_ only we obtain log-likelihood as a function of the number of channels:

(16) $$\begin{array}{l} -\log{\text{L}}_{\theta }\left(c_{i}-n_{i}\right)=\frac{1}{2}\left(\frac{c_{i}^{\text{T}}c_{\text{m}1}^{-1}c_{i}-n_{i}^{\text{T}}c_{\text{m}1}^{-1}n_{i}}{{\text{N}}_{\text{chi}}}+\mu^{\text{T}}c_{\text{m}1}^{-1}\mu {\text{N}}_{\text{chi}}\right)\\ \;+\frac{{\text{N}}_{\text{T}}}{2}\log{\text{N}}_{\text{chi}} \end{array}$$

The number of channels, N_chi_, can be approximated for each macroscopic synaptic current ***c***_*i*_ as a number that gives maximum of the likelihood function when being substituted into Equation (16). Therefore, after differentiation of Equation (16) with respect to N_ch_

(17) $$\begin{array}{l} \frac{\partial \log{\text{L}}_{\theta }\left(c_{i}-n_{i}\right)}{\partial {\text{N}}_{\text{chi}}}=0=\frac{1}{2{\text{N}}_{\text{chi}}^{2}}{\left(c_{i}-n_{i}\right)}^{\text{T}}c_{\text{m}1}^{-1}\left(c_{i}-n_{i}\right)-\frac{{\text{N}}_{\text{T}}}{2{\text{N}}_{\text{chi}}}\\ \;-\frac{1}{2}\mu^{\text{T}}c_{\text{m}1}^{-1}\mu \Rightarrow {\text{N}}_{\text{chi}}^{2}\mu^{\text{T}}c_{\text{m}1}^{-1}\mu +{\text{N}}_{\text{chi}}{\text{N}}_{T}\\ \;-{\left(c_{i}-n_{i}\right)}^{\text{T}}c_{\text{m}1}^{-1}\left(c_{i}-n_{i}\right)=0 \end{array}$$

we find an approximation for the number of channels, N_chi_, for each macroscopic synaptic current

(18) $${\text{N}}_{{\text{ch}}_{i}}=\frac{-{\text{N}}_{\text{T}}+\sqrt{{\text{N}}_{\text{T}}^{2}+4c_{i}^{\text{T}}c_{\text{m}1}^{-1}c_{i}\cdot \mu^{\text{T}}c_{\text{m}1}^{-1}\mu }}{2\mu^{\text{T}}c_{\text{m}1}^{-1}\mu }$$

Here ***c**_i_* is not the whole decaying phase of each current but only those part where signal-to-noise ratio is high and therefore noise term can be neglected (usually from peak of the current to 0.1–0.5 of the peak). Therefore, before calculating the log-likelihood, we first estimate N_chi_ for each macroscopic synaptic current, ***c**_i_*, then substitute N_chi_ into Equations (13) and (14) and calculate the log-likelihood of the set of parameters θ given the set of simulated macroscopic synaptic currents. Accordingly, N_ch_ is estimated automatically when the maximization is finished.

The peak open probability, P(o, peak), was defined as a probability that a channel is opened at the peak of the macroscopic current given that this channel was bound with a neurotransmitter immediately after the end of concentration transient, which was assumed to be sufficiently short (0.1–0.2 ms) with respect to the time interval (1–4 ms) from the moment of stimulation to the starting point of the analyzed fragment of current. P(o, peak) can be expressed as a function of rate constants: P(o, peak) = max(e^**Qt**^***p***(0)), where ***p*** (0) is an initial state probability vector assumed to be zero for all states except for the RG2 state in the case when currents were simulated with 7-state GABA_A_R scheme or RL state in the case when currents were simulated with simple 3-state kinetic scheme (see descriptions of both schemes below).

Summing up, ML NSFA can be used for the fast estimation of rate constants, unitary current of synaptic ion channel, the number of synaptic channels bound with a neurotransmitter right after the concentration transient for each synaptic current and peak open probability from the set of macroscopic synaptic currents under Gaussian colored background noise.

### The log-likelihood maximization procedure

We search for the log-likelihood global maximum to obtain the required model parameters from a set of macroscopic synaptic currents. In order to do this, we minimize the negative log-likelihood with a variant of graduated optimization technique using SQP algorithm embedded in *fmincon* function in MATLAB Optimization toolbox. Initial estimates of each parameter were chosen randomly and uniformly from the logarithmic scale interval, [θ_0_/10, θ_0_ · 10], where θ_0_ is a vector composed of the true values of each parameter (rate constants and unitary current), i.e., of values utilized by the macroscopic current generator (see below). During the search of a minimum, all parameters were bounded within the interval [θ_0_/50, θ_0_ · 50].

In our version of graduated optimization technique, the whole minimization procedure was divided into sequential minimization steps. On the first step the negative log-likelihood was minimized given the first 2 or 3 currents regularly sampled at 50 points each. On each consequent minimization step the number of points and the number of currents was increased. The parameter estimates, θ_*ML*_, obtained on each previous step were taken as initial parameters θ_0_ for each next minimization step. For all calculations in this work each minimization was rerun 5 (3-state scheme) or 10 (7-state scheme) times, each time starting from the different initial parameter set.

### Simulation of macroscopic synaptic currents

First series of simulations of macroscopic synaptic currents were based on experimentally derived 7-state kinetic scheme for GABA_A_ receptor that had one unliganded state, R, two liganded closed states (RG, singly-liganded and RG2, doubly-liganded) and the respective open (O1 and O2) and desensitized (D1 and D2) states (Mozrzymas et al., 2003). The following rate constants were adapted from Mozrzymas et al. (2003): *k_off_* = 0.13 ms^−1^, *d*_1_ = 0.14 ms^−1^, *d*_2_ = 1.5 ms^−1^, *r*_1_ = 0.02 ms^−1^, *r*_2_ = 0.12 ms^−1^, *a*_1_ = 1.5 ms^−1^, *a*_2_ = 1 ms^−1^, *b*_1_ = 0.15 ms^−1^, *b*_2_ = 8 ms^−1^; *k*_*on*1_ = 4 ms^−1^ mM^−1^, *k*_*on*2_ = 8 ms^−1^ mM^−1^; Unitary current was the same for singly- and doubly-liganded states and was set to 1 pA. Variability in the amplitude of macroscopic postsynaptic responses was achieved by trail-to-trial Gaussian variation of the number of available synaptic channels (mean = 250; *SD* = 50). Simulation time step was Δ*t* = 0.2 ms. Synaptic vesicle release was modeled as a square pulse of saturating agonist concentration with a duration equal to the single simulation time-step (Δ*t* = 0.2 ms), which caused transition of all available channels from R to RG2 state. A total of 1000 macroscopic synaptic currents were simulated and colored noise that resembled baseline noise of experimentally recorded IPSCs was added to each current. Colored noise (*SD* = 3 pA) was modeled as a sum of 4 AR(1) processes (Qin et al., 2000; Venkataramanan and Sigworth, 2002):

(19) $$\begin{array}{l} noise_{t}=\sum_{k=1}^{N_{noise}}noise_{t,k},noise_{t,k}=\varphi_{\text{k}}noise_{(\tau -1),k}\\ \;+\sigma_{k}{\text{w}}_{t,k},{\text{w}}_{t,k}\sim \text{N}(0,1) \end{array}$$

with parameters ϕ = [0.0067, 0.61, 0.96, 0.999] and **σ** = [0.32, 1.0, 1.42, 0.72], pA that were obtained from the approximation of autocorrelation function of experimentally recorded (whole-cell patch clamp) background noise by the sum of 4 exponentials (see Equations 23, 24 in Stepanyuk et al., 2011). The decaying phases of the responses (starting in 1 ms after the end of stimulation pulse) were taken for the consequent log-likelihood maximization.

In a second series of simulations we have used simple 3-state kinetic scheme of an abstract synaptic receptor. The scheme consisted of unliganded state, R, singly-liganded state, RL, and open state, O and had the following rate constants: binding rate, *k*_on_ = 6 mM^−1^ms^−1^, unbinding rate, *k*_off_ = 0.025 ms^−1^, opening rate, *b* = 0.25 ms^−1^. The closing rate constant, *a*, was 2.5 ms^−1^ for Model R and Model N and 1.25 ms^−1^ for Model A (see Section ML NSFA Distinguishes Between Changes in the Channel Gating and Changes in the Number of Receptors Bound with a Neurotransmitter in Results). Unitary current was set to 1 pA. Variability in the amplitude of macroscopic postsynaptic responses was achieved by trail-to-trial Gaussian variation of the number of available synaptic channels (mean = 400; *SD* = 50 for Models R and A; mean = 800; *SD* = 71 for Model N). Simulation time step was Δ*t* = 0.1 ms. Synaptic vesicle release was modeled as a square pulse of saturating agonist concentration with a duration equal to two simulation time-steps (0.2 ms), which caused transition of all channels from R to RL state.

### Accuracy of the estimates

Accuracy of kinetic rates, unitary current, number of liganded channels, and peak open probability estimates was estimated using bootstrap. To this end, *N* = 5, 10, 20, 30, 40, or 100 currents were randomly sampled with replacement from the initially generated set of 1000 macroscopic current traces. This procedure was repeated until that 30–40 bootstrap samples were generated. For each bootstrap sample we rerun likelihood maximization *m* = 5 or 10 times (for currents generated with 3- or 7-state scheme, respectively), starting m-1 times from different randomly generated initial parameter sets (see Section The Log-Likelihood Maximization Procedure above) and mth start was done from θ_0_. The estimated model parameters, **θ**_***ML***_, were obtained from a maximization trial that resulted in the best log-likelihood, which was considered to be a global maximum. The accuracy of estimated model parameters was assessed as a deviation of these parameters (θ*_ML_*) from those (θ_0_) used for the generation of the currents, $\frac{\sqrt{{\left(\theta_{ML}-\theta_{0}\right)}^{2}}}{\theta_{0}}$ (hereinafter relative error). The algorithm was implemented in MATLAB.

### Peak-scaled non-stationary fluctuation analysis

Accuracy of single-channel current estimates obtained with ML NSFA method presented here was compared to those obtained by PS NSFA. In PS NSFA, variance in currents arising from the stochastic gating of the ion channel is isolated from variance arising from sources such as quantal variability by scaling the mean simulated current waveform to the peak amplitude of each individual simulated current and then subtracting the two waveforms (Traynelis et al., 1993).

(20) $$I_{i}^{peak-scaled}=I_{i}-\langle I\rangle \frac{\text{max}\left(I_{i}\right)}{\text{max}\langle I\rangle }$$

To estimate the accuracy of unitary current estimates with PS NSFA, it was applied to *n* = 1000 bootstrap samples each of which contained either *N* = 5, 10, 20, 30, 40, or 100 currents simulated with a 7-state GABA_A_ receptor scheme (see Section Simulation of Macroscopic Synaptic Currents above). For each bootstrap sample the ensemble variance, σ^2^, was plotted against the ensemble mean, 〈*I*〉, and then fitted with parabola:

(21) $$\sigma^{2}\left(I^{peak-scaled}\right)=i_{ch}\langle I\rangle -\frac{{\langle I\rangle }^{2}}{\langle {\text{N}}_{\text{ch}}\rangle }+\sigma_{0}^{2}$$

where σ^2^_0_ is the variance of the background noise. Accuracy of unitary current estimates was calculated as described above, and was then compared with the accuracy of estimates obtained with ML NSFA. To ensure the best accuracy possible with PS NSFA, the ensemble mean current 〈*I*〉 and variance, σ^2^, were calculated for each data point and the rising phase of variance *vs*. mean curve was fitted with parabola using weighted (with weights ω*_i_* = 1/var(σ^2^*_i_*)) least squares method.

### Estimation of unitary current from a single macroscopic current

Sampling from a likelihood distribution of model parameters that were estimated from a single macroscopic synaptic current was done by the slice sampling Markov chain Monte Carlo algorithm (Neal, 2003), implemented in “MCMC Methods for MLP and GP and Stuff” toolbox by Toni Auranen and Aki Vehtari (available at http://www.lce.hut.fi/research/compinf/mcmcstuff/).

## Results

### ML NSFA applicability to estimation of unitary current and kinetic constants of postsynaptic receptor channels

Postsynaptic architecture restricts direct electrophysiological access to individual receptors in native synaptic environments, with only occasional exceptions when channel openings and closings can be resolved on the very tail of postsynaptic currents (Silver et al., 1992). Both these exceptions and application of PS NSFA (Traynelis et al., 1993) do not allow estimating any parameters of synaptic receptors except their unitary conductance and the number of receptors open at the peak of synaptic current (Hartveit and Veruki, 2006).

**Figure 1:**
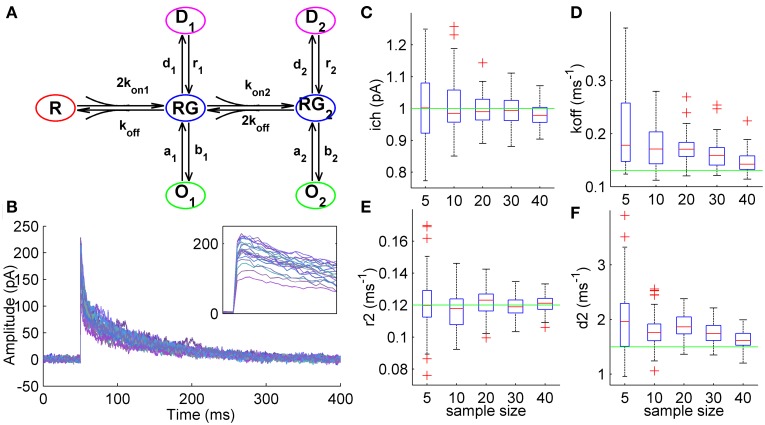
**Estimation of unitary current and kinetic constants from simulated GABAergic synaptic currents. (A)** 7-state kinetic scheme of GABA_A_ receptor that was used to simulate macroscopic synaptic currents (Mozrzymas et al., 2003, see Section Simulation of Macroscopic Synaptic Currents in Methods). The scheme has one unbound state, R, two liganded states (single-liganded, RG, and double-liganded, RG2,) and related open (O1 and O2) and desensitized (D1 and D2) states. Rate constants were adapted from Mozrzymas et al. (2003) and were as follows: *k*_off_ = 0.13 ms^−1^, *d*_1_ = 0.14 ms^−1^, *d*_2_ = 1.5 ms^−1^, *r*_1_ = 0.02 ms^−1^, *r*_2_ = 0.12 ms^−1^, *a*_1_ = 1.5 ms^−1^, *a*_2_ = 1 ms^−1^, *b*_1_ = 0.15 ms^−1^, *b*_2_ = 8 ms^−1^; *k*_*on*1_ = 4 mM^−1^ ms^−1^, *k*_*on*2_ = 8 mM^−1^ ms^−1^. Unitary currents for the states O1 and O2 were equal and were set to *i*_1_ = *i*_2_ = 1 pA. The number of channels exposed to GABA varied from trial to trail (N_ch_ = 250, *SD* = 50; Gaussian variation). Colored noise (*SD* = 3 pA) was added to the simulated currents (see Section Simulation of Macroscopic Synaptic Currents in Methods). **(B)** Synaptic currents simulated using the kinetic scheme shown in **(A)**. The currents demostrate high trial-to-trial variability resembling one observed in experimental electrophysiological recordings (inset). **(C)** Statistical plots demonstrating accuracy of unitary current estimates obtained by ML NSFA. On each plot, the central mark (red) is the median, the edges of the box are the 25th and 75th percentiles, the whiskers extend to the most extreme data points not considered outliers, and outliers are plotted individually by red crosses. Green line indicates true value of unitary current (1 pA). Note high accuracy of unitary current estimates obtained by ML NSFA even if a few (5–20) currents were used. **(D–F)** Statistical plots of estimates of some kinetic constants obtained by ML NSFA. Colors are the same as in **(C)**.

In this part of the work we tested how ML NSFA estimates the unitary current and kinetic constants of GABA_A_ receptors from stochastically simulated macroscopic currents having a trial-to-trial Gaussian variation in the number of available receptors (N_ch_ = 250 ± 50). Currents were simulated with a 7-state model of this receptor (Mozrzymas et al., 2003, see Methods) having one unbound, two liganded closed, two open and two desensitized states (Figure 1A). Synaptic release of GABA was modeled as a brief (0.2 ms) step of saturating GABA concentration resembling GABA release in real synaptic connections (Perrais and Ropert, 1999, 2000; Hájos et al., 2000; Nusser et al., 2001; Biró et al., 2006; Scimemi and Beato, 2009). 1000 macroscopic currents generated in response to this stimulation had the mean amplitude of 184 ± 35 pA and decay kinetics of 43.4 ± 3.6 ms (Figure 1B) and resembled postsynaptic currents routinely recorded in cortical GABAergic synapses (Nadkarni et al., 2010). Background colored noise (*SD* = 3 pA, see Section Simulation of Macroscopic Synaptic Currents in Methods) was added to the simulated currents.

Samples consisting of *N* = 5, 10, 20, 30, or 40 macroscopic currents were randomly chosen from initially generated set of 1000 currents and analyzed using ML NSFA. In order to assess the accuracy of estimates for the unitary current and kinetic rates, parameter search was performed for 60 samples obtained in such a way and log-likelihood maximization was run 10 times for each sample in order to achieve the global maximum (see Section Accuracy of the Estimates in Methods). For each run, the initial parameter values were chosen randomly and uniformly in the logarithmic scale from the range [θ_0_/10, θ _0_ · 10], where θ_0_ denotes true parameter values, i.e., those used for simulation of currents.

The unitary current was estimated with good accuracy even from samples consisting of only 10 simulated postsynaptic currents (Figure 1C, 8.1% relative error) whereas it was estimated with almost 2-fold better accuracy when the number of currents in the sample was increased from 10 to 40 (4.3% relative error). Three rate constants: unbinding rate (*k*_off_), desensitization (*d*_2_) and resensitization (*r*_2_) rate from double-liganded state could also be estimated (Figures 1D–F). For samples consisting of 10 and 40 currents the relative errors of estimates were: *k_off_* —49.0% and 19.1%; *d*_2_—28.3 and 14.6%; *r*_2_—8.9 and 4.7%, respectively. Some of rate constants associated with single-liganded states were estimated in order of magnitude (*a*_1_) or bounded from below (*b*_1_, *d*_1_).

Thus, we demonstrate that ML NSFA could reliably estimate the unitary current of synaptic receptor channel and several kinetic constants of synaptic receptor model from the very limited number of postsynaptic currents (5–40). These results indicate that ML NSFA may allow analysis of kinetic models of synaptic receptors in their native biochemical environment using routinely recorded macroscopic postsynaptic currents.

### ML NSFA accuracy in estimation of unitary current compared to PS NSFA

The number of currents necessary for a particular algorithm to secure a given accuracy of unitary current estimate is an important practical issue. With many hundreds or even thousands of simulated macroscopic currents accuracy of PS NSFA in estimating the unitary current is fairly good (Markova et al., 2005; Hartveit and Veruki, 2006). At the same time it is hard to collect more than about 100 of evoked postsynaptic currents in steady-state conditions in routine electrophysiological experiments.

Thus, to see whether ML NSFA gives any advantage with respect to the number of required traces we calculated a relative error of unitary current estimates obtained with ML NSFA from the above described samples of different sizes (5, 10, 20, 30, 40, and 100 currents; 60 samples were analyzed in each case to estimate the error) and compared this error with one estimated with PS NSFA applied to 1000 samples of similar sizes.

**Figure 2:**
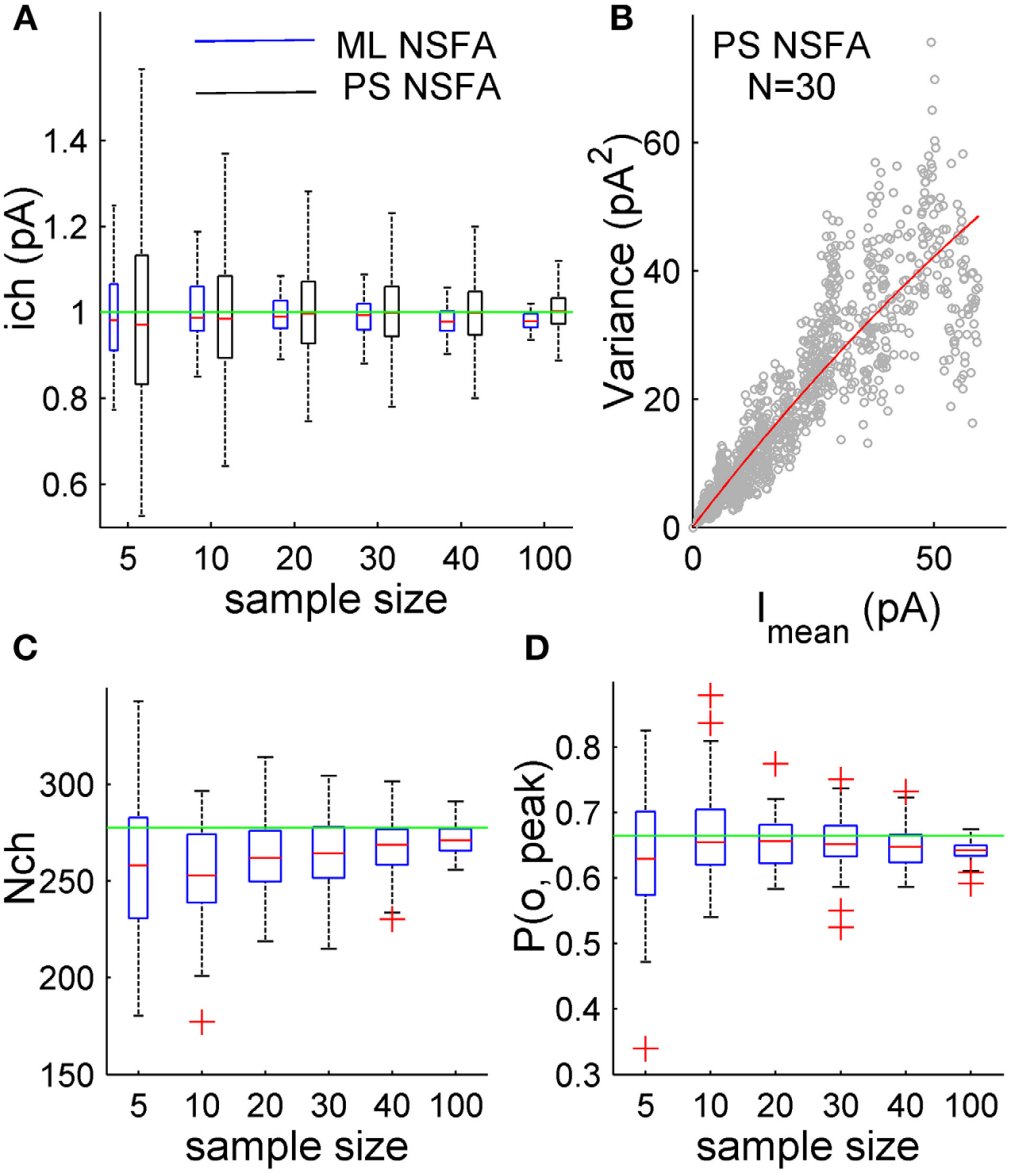
**ML NSFA is more accurate than PS NSFA in estimating of unitary current**. Estimation of the number of receptors bound with a neurotransmitter and peak open probability with ML NSFA. **(A)** Statistical plots demonstrating accuracy of unitary current estimates obtained with ML NSFA (blue boxes) and PS NSFA (black boxes) from simulated macroscopic synaptic currents with trial-to-trial Gaussian variation in the number of receptors (N_ch_ = 250, *SD* = 50; see kinetic scheme in Figure 1A). On each plot, the central mark (red) is the median, the edges of the box are the 25th and 75th percentiles, the whiskers extend to the most extreme data points not considered outliers, and outliers are plotted individually by red crosses. Green line indicates a true value of unitary current. ML NSFA and PS NSFA were performed using *n* = 60 and *n* = 1000 samples consisting of *N* = 5, 10, 20, 30, 40 or 100 simulated currents, respectively. Note that the accuracy of estimates obtained with ML NSFA using a few (5–20) currents was 2-times better than one obtained with PS NSFA. **(B)** An example of variance *vs*. mean plot (gray dots) obtained with PS NSFA for *N* = 30 simulated macroscopic currents having trial-to-trial Gaussian variation in the number of receptors (N_ch_ = 250, *SD* = 50) and a parabolic fit of its rising phase (red). Note that variance-mean relationship (gray dots) is skewed rather than parabolic and therefore the number of receptors could not be estimated with PS NSFA. **(C,D)** Statistical plots for the estimates of the number of channels bound with a neurotransmitter right after the concentration transient, N_ch_, and peak open probability, P(o, peak) obtained with ML NSFA. Green line in **C** indicated the true value of the number of channels estimated as mean peak current amplitude (averaged over *N* = 1000 currents) divided by the true value of P(o, Peak) and by the true value of unitary current (1 pA) and in **D** green line indicates the true value of P(o, Peak) estimated as P(o) = e^Qt^*p*_0_. Other colors and notations are the same as in Figure 1C.

Figure 2A demonstrates that the error of unitary current estimates obtained with both methods decreases with the number of currents taken for the analysis. However, the unitary current can be estimated with as low as 10.8, 8.1, and 4.9% relative error from only 5, 10, and 20 simulated synaptic currents, respectively, whereas PS NSFA resulted in about 2-fold lower accuracy (23.0, 14.7, and 10.4 relative error, respectively). The estimates obtained with ML NSFA from the analysis of samples of 30 and 40 currents had relative error of 4.6 and 4.3%, whereas PS NSFA gave 8.6 and 7.2% error for these cases. At last, accuracies of unitary current estimates obtained from 100 simulated currents were high for both methods and were comparable (Errors: 2.9% for ML NSFA vs. 4.5% for PS-NSFA; Figure 2A).

Thus, for some complex models ML NSFA allows evaluation of the unitary current with good accuracy using experimentally realistic number of macroscopic currents and substantially outperforms PS NSFA in terms of accuracy when only a few (5–30) postsynaptic currents are available for estimating the unitary current.

### ML NSFA estimates the number of synaptic receptors bound with neurotransmitter and peak open probability

PS NSFA was specifically designed to be independent of variations in the number of postsynaptic receptors exposed to neurotransmitter and peak open probability for the sake of more accurate estimation of a unitary current (Silver et al., 1996) from postsynaptic current fluctuations. Unfortunately, this method could not be used for the estimation of the total number of receptors in the synapse. To the contrary, ML NSFA presented here allows estimation of the number of receptors bound with neurotransmitter by the end of neurotransmitter concentration transient in each macroscopic current (liganded channels, N_ch_). It is assumed that this transient time course is known or sufficiently brief, meaning that it could be approximated by delta function in the latter case. Indeed, GABA concentration in the synaptic cleft decreases by a factor of 10 during less than 0.1 ms after synaptic vesicle release (Scimemi and Beato, 2009) resulting in almost instantaneous concentration transient. For such a brief concentration transient and for a given GABA receptor model (Figure 1A) ML NSFA would estimate the number of receptors bound with two GABA molecules by the end of concentration transient in all synapses of particular synaptic connection independently upon receptor saturation in the case when most of the current is mediated by the receptors in double-liganded states.

The open probability P(o) at any given time is determined as a mean current divided by a product *i*_ch_N_ch_, and it is a function of rate constants: P(o) = e^**Qt**^***p***(0) (see Section Estimation of the Number of Channels and Peak Open Probability in Methods). Thus, P(o) as a function of time is automatically estimated at the end of log-likelihood maximization procedure. The peak open probability is simply a maximum of this function, P(o, peak) = max (e^**Q*t***^***p***(0)). Peak open probability estimated by ML NSFA is, thus far, a ratio of the number of receptors open at the peak of postsynaptic current to the number of double-liganded receptors by the end of neurotransmitter concentration transient.

Figures 2C,D demonstrate that the error of N_ch_ and P(o, peak) estimates obtained with ML NSFA decreases with the number of currents taken for the analysis. The number of liganded receptor channels, N_ch_, was calculated as an average over all currents in the sample and was estimated with 24.5 and 12.4% relative error from samples consisting of only 5 and 10 simulated macroscopic synaptic currents, respectively. The respective estimates of accuracy for the peak open probability, P(o, peak), had 14.4 and 9.8% relative error, respectively. Both N_ch_ and P(o, peak) were estimated with even better accuracy from samples consisting of 100 simulated currents (10.0 and 4.3% relative error, respectively).

At the same time PS NSFA applied to the same samples resulted in a variance vs. mean curve that was profoundly skewed (Figure 2B, gray dots) and, therefore, could not give an estimate of the number of liganded channels, N_ch_.

### Estimation of unitary current and kinetic constants of receptors having multiple conductance levels

Most ligand-gated channels are described by kinetic schemes with multiple, non-identical open states often having different conductance levels (Jin et al., 2003; Mozrzymas et al., 2003; Robert and Howe, 2003; Wyllie et al., 2006; Keramidas and Harrison, 2010; Mortensen et al., 2010). In practice some open states should be considered rare and excluded from the fitting of experimental results in order to estimate at least some parameters of receptor kinetic schemes (Mortensen et al., 2010). Unfortunately, PS NSFA is also not applicable to examination of receptors having multiple conductance levels giving values of unitary current and channel number having no obvious physical interpretation (Hartveit and Veruki, 2006). Thus, at the present moment single-channel recordings are virtually the only approach that allows identifying multiple conductance levels of ligand-gated receptors and this approach is also not applicable for studying of synaptic receptors.

**Figure 3:**
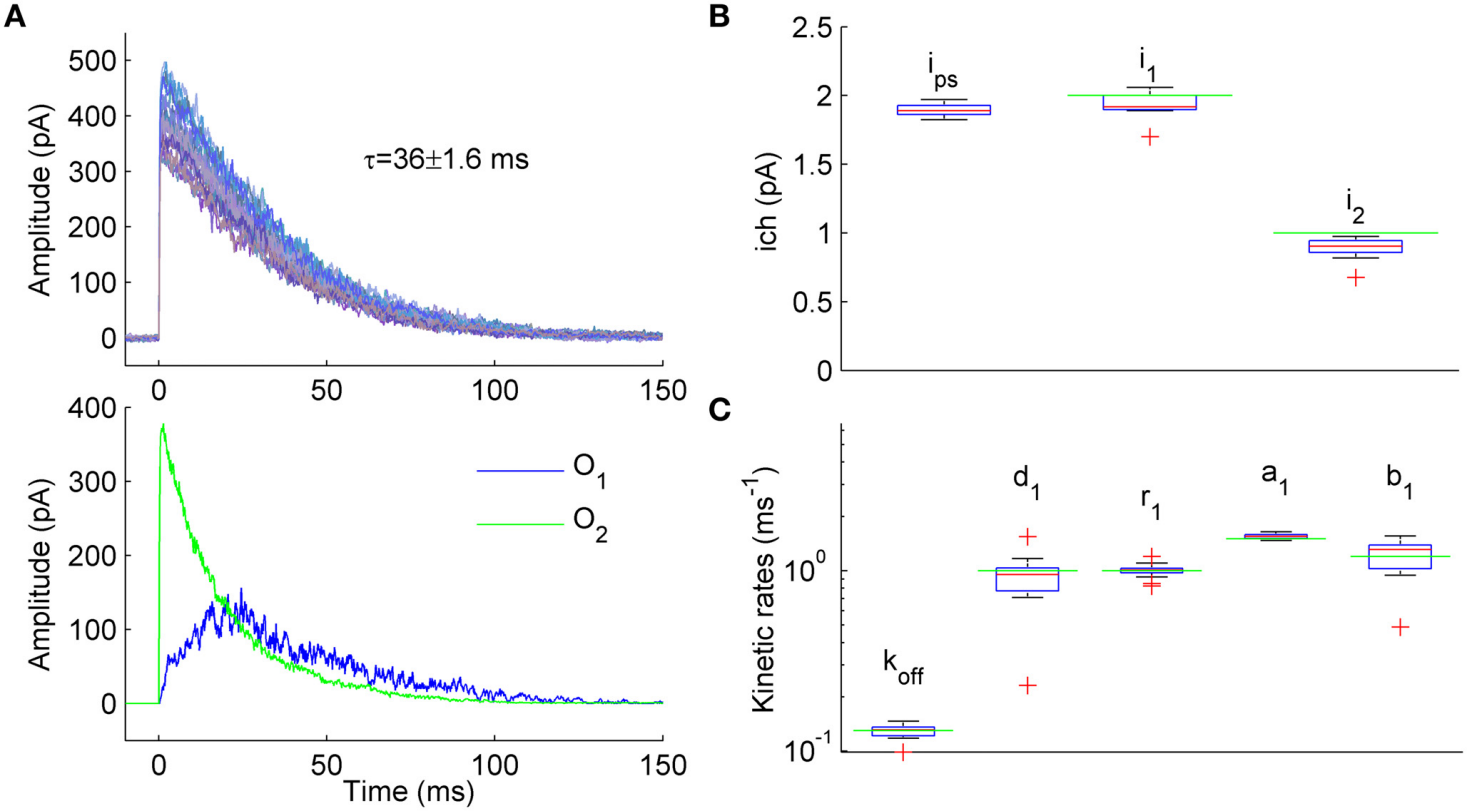
**Estimation of unitary currents and kinetic constants of receptors having two open states with different conductance levels. (A)** *Upper panel*. Example of 50 synaptic currents simulated with a 7-state kinetic scheme of GABA_A_ receptor having two open states (Figure 1A, some rate constants were modified: *b*_2_ = 4, *b*_1_ = 1.2, *d*_1_ = 1, *r*_1_ = 1, *d*_2_ = 0.15, *r*_2_ = 1 ms^−1^). Unitary currents were set to *i*_1_ = 2 pA and *i*_2_ = 1 pA for open states O1 and O2, respectively. The number of channels varied from trial to trail (N_ch_ = 500 ± 50; Gaussian variation). *Lower panel*. Representative example of single simulated macroscopic current components mediated by single-liganded open state O1 (blue trace) and double-liganded open state O2 (green trace) demonstrating comparable contribution of O1 and O2 to the total macroscopic current. **(B)** Statistical plots for the estimates of unitary currents obtained with PS NSFA (leftmost bar, *i* = 1.86 ± 0.03 pA) and ML NSFA (two bars on the right, *i*_1_ = 2.0 ± 0.11 pA and *i*_2_ = 0.89 ± 0.08 pA, *i*_1_ and *i*_2_ are unitary currents associated with open states O1 and O2, respectively. Both PS NSFA and ML NSFA were applied to samples of 50 macroscopic currents (*n* = 15 and *n* = 250 bootstrap samples for MS NSFA and PS NSFA, respectively) simulated as described in **(A)** and having true values of *i*_1(0)_ = 2 pA and *i*_2(0)_ = 1 pA, respectively (indicated by green lines). On each plot, the central mark (red) is the median, the edges of the box are the 25th and 75th percentiles, the whiskers extend to the most extreme data points not considered outliers, and outliers are plotted individually by red crosses. Note that ML NSFA accurately distinguishes both unitary current levels, whereas PS NSFA gave some value of the unitary current that was close to *i*_1(0)_. **(C)** Statistical plot for the estimates of kinetic rates of transitions from and to a single-liganded state obtained by ML NSFA (in ms^−1^: unbinding rate, *k*_off_ = 0.13 ± 0.01, desensitization rate, *d*_1_ = 0.89 ± 0.34, resensitization rate *r*_1_ = 1.02 ± 0.08, closing rate, *a*_1_ = 1.55 ± 0.05, opening rate, *b*_1_ = 1.17 ± 0.24; *N* = 50 currents simulated as described in **(A)**. The estimates were in good agreement with their true values (green lines). See a legend to panel **(B)** for further description.

We next wanted to investigate if ML NSFA suggested in this work is applicable to analysis of ion channels and ligand-gated receptors with multiple conductance levels, described by kinetic schemes with non-identical open states. 7-state kinetic model of GABA_A_ receptor (Mozrzymas et al., 2003) having two open states O1 and O2 with identical unitary current (*i*_1_ = *i*_2_, see Figure 1A) was modified to have the unitary current *i*_1_ = 2 pA and *i*_2_ = 1 pA for the states O1 and O2, respectively (Figure 3). Rate constants of the model were modified in such a way that the contribution of single- and double-liganded open states to the total macroscopic current became comparable. Modified constants were (in ms^−1^): *b*_2_ = 4, *b*_1_ = 1.2, *d*_1_ = 1, *r*_1_ = 1, *d*_2_ = 0.15, *r*_2_ = 1. Colored background noise with *SD* = 3 pA was added to the simulated currents (Figure 3A, upper panel).

Representative examples of the simulated current components associated with either state O1 or state O2 are shown in Figure 3A, lower panel, by blue and green lines, respectively. When 250 samples consisting of *N* = 50 simulated currents (Figure 3A, upper panel) were analyzed by PS NSFA the unitary current estimates were close to the unitary current of single-liganded open state O1 (1.86 ± 0.03 pA *vs i*_1_ = 2 pA for the state O1). At the same time, ML NSFA gave reasonable estimates for both conductance levels (Mean ± *SE i*_1_ = 2.00 ± 0.11 pA and *i*_2_ = 0.89 ± 0.08 pA; *n* = 15 samples of *N* = 50 simulated currents; Figure 3B). ML NSFA also reliably estimated kinetic rates for single-liganded state transitions (*k*_off_ = 0.13 ± 0.01, *d*_1_ = 0.89 ± 0.34, *r*_1_ = 1.02 ± 0.08, *a*_1_ = 1.55 ± 0.05, *b*_1_ = 1.17 ± 0.24 ms^−1^, Figure 3C) and the mean number of liganded channels (N_ch_ = 557 ± 53 vs. 500 ± 50 used in simulation).

Thus, contrary to PS NSFA, ML NSFA can reliably estimate kinetic schemes with several open states having different conductance levels and gives precise values of unitary currents, some kinetic rates, and the mean number of liganded receptors in a given synaptic connection.

### ML NSFA distinguishes between changes in the channel gating and changes in the number of receptors bound with a neurotransmitter

We next attempted to explore ML NSFA capability to identify which postsynaptic parameters were changed in the case when mean amplitude of simulated currents was increased without changes in macroscopic current waveform and unitary current.

**Figure 4:**
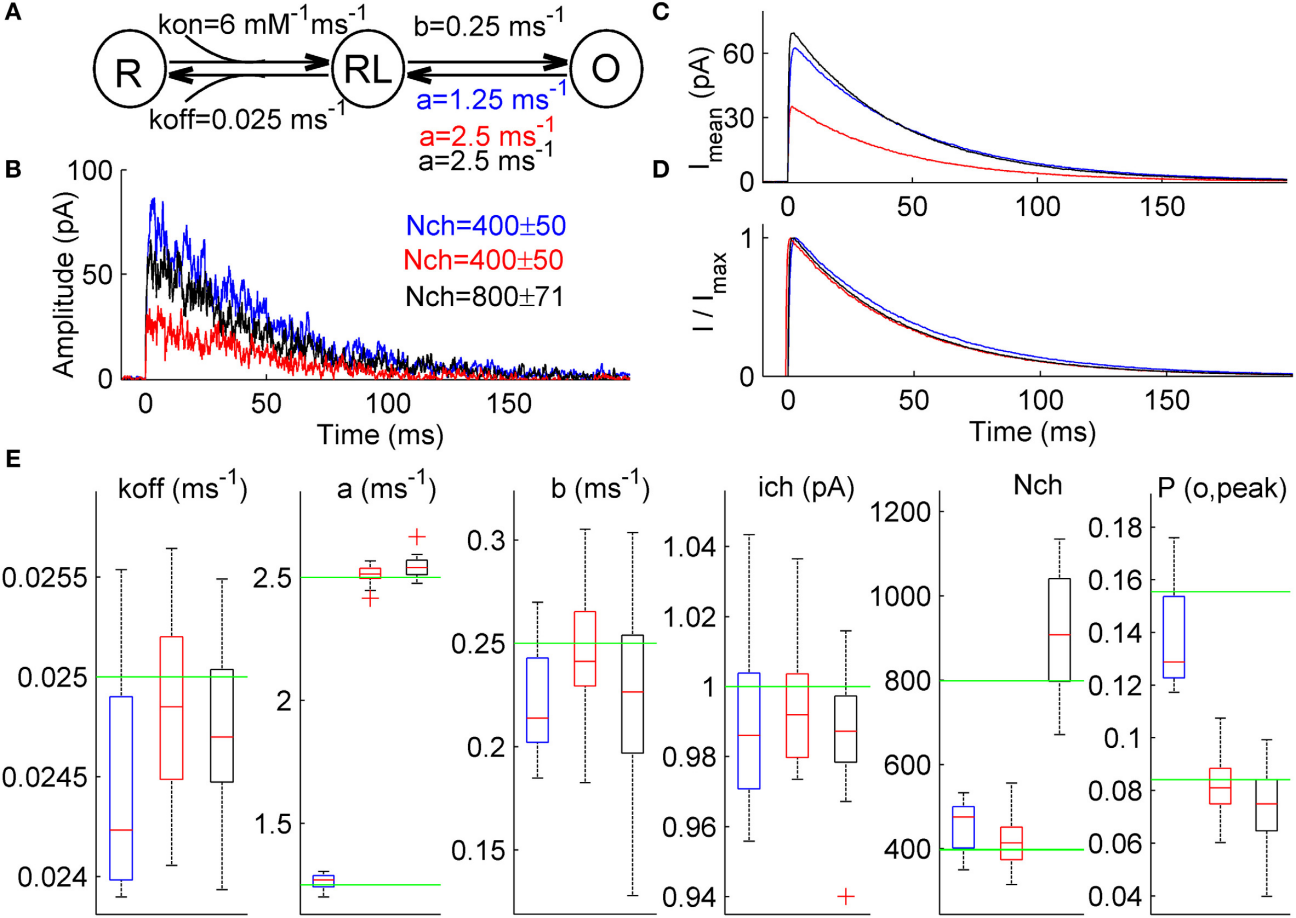
**ML NSFA distinguishes between changes in the receptor gating and the number of receptors in case when both unitary current and macroscopic current waveform are not changed. (A)** Simple 3-state kinetic scheme of synaptic receptor channel. The scheme consists of one unbound state, R, one single-liganded state, RL, and one open state, O. Rate constants are shown below the respective transitions and were as follows: *k*_on_ = 6 mM^−1^ ms^−1^, *k*_off_ = 0.025 ms^−1^, *b* = 0.25 ms^−1^. Three different models were constructed based on this scheme and were used to simulate 3 sets of macroscopic currents. A closing rate constant, *a*, was set to 2.5 ms^−1^ for Model R (red) and Model N (black) and 1.25 ms^−1^ for Model A (blue). **(B)** An example of 3 macroscopic currents simulated using Model R (red), Model A (blue), and Model N (black) shown in **(A)**. The number of channels used for simulations is indicated in a respective color in the top-right corner (400 ± 50 for Models R and A and 800 ± 71 for Model N). **(C)** Mean simulated currents for each Model (*N* = 1000). Note that amplitudes of mean currents obtained with Model A and Model N (blue and black) are almost equal and almost twice larger than the mean current amplitude obtained with a reference Model R (red). **(D)** The same mean currents as in **(C)** but normalized. Note that all 3 waveforms almost coincide. **(E)** Statistical plots for the estimates of kinetic rates, unitary current, number of channels bound with a neurotransmitter, N_ch_, and peak open probability, P(o, peak), obtained with ML NSFA (*N* = 100 currents; *n* = 20 bootstrap samples). On each plot, the central mark (red) is the median, the edges of the box are the 25th and 75th percentiles, the whiskers extend to the most extreme data points not considered outliers and outliers are plotted individually by red crosses. Green line indicates true value of parameter. Blue, red, and black boxes correspond to results of ML NSFA applied to macroscopic currents generated with Model A, R, and N, respectively. Note that estimates for the closing rate, *a*, and peak open probability, P(o, peak), obtained from currents generated with Model A (blue boxes) are close to their true values and do not coincide within SE's with the respective estimates obtained for reference Model R (red boxes). At the same time, estimate for the number of channels, N_ch_, obtained from currents generated with Model N is close to its true value and differs from the respective value obtained from currents generated with reference Model R.

To this end three distinct groups of 1000 macroscopic currents were generated using a simple 3-state scheme of synaptic channel (Figure 4A, see Section Simulation of Macroscopic Synaptic Currents in Methods). A similar increase in mean current amplitude was achieved by changes in either receptor gating or receptor number. A reference kinetic scheme (Model R; Figure 4A, red) had the closing rate, *a* = 2.5 ms^−1^ and the total number of channels N_ch_ = 400 ± 50 and was used to generate a group of macroscopic currents before putative remodeling of synaptic connection (Figure 4B). In the second kinetic scheme (Model A; Figure 4A, blue) mimicking remodeling of receptor gating the closing rate, *a*, was changed from 2.5 ms^−1^ to 1.25 ms^−1^ resulting in almost 2-fold increase of average current amplitude (Figure 4C, blue) without substantial changes in current waveform (Figure 4D, blue vs. red). Conversely, in the third model (Model N; Figure 4A, black) the number of available channels, N_ch_, was increased from 400 ± 50 to 800 ± 71 without any changes in the kinetic constants, which led to similar changes in current amplitude (Figure 4C, black) as for Model A without any changes in current waveform (Figure 4D, black vs. red). Therefore, currents generated with Models A and N had similar amplitudes and when normalized, appeared to have the same waveforms as reference currents generated by Model R (Figures 4B–D).

ML NSFA was run with *n* = 20 bootstrap samples consisting of *N* = 100 currents (see Section Accuracy of the Estimates in Methods) for each of the 3 groups of simulated currents in order to evaluate the receptor model parameters and the respective errors. Log-likelihood maximization was run 5 times for each bootstrap sample in order to achieve the global maximum. When the parameter estimates obtained from currents generated with Model R were compared to those obtained from currents generated with Model A (Figure 4E, red vs. blue boxes) the difference, Δ_RA_, between mean values of each parameter estimates except the closing rate, *a*, and peak open probability, P(o, peak), was small and was within the standard error (*SE*) range of the respective estimates: *k*_off_: Δ_RA_ = 1.7% (*SE* = 2.2%), *b*: 8.5% (13.0%), *i*_ch_: 0.6%(2.4%), N_ch_: 8.8% (14.0%). At the same time, Δ_RA_ was 49.7% for the closing rate, *a* and 70.1% for the peak open probability, P(o, peak) and did not fall within the narrow ranges of the respective *SE*'s (2.4% and 14.1%, respectively). The mean values of the respective estimates were *a* = 2.51 ± 0.04 for Model R and 1.26 ± 0.03 for Model A, P(o, peak) = 0.08 ± 0.01 for Model R and 0.14 ± 0.02 for Model A. Therefore, we could infer that these were the parameters that altered. These results directly indicate that ML NSFA may reliably determine changes in receptor gating, which leads to an increase in peak open probability.

When estimates obtained from currents generated with Model R and Model N were compared, we observed insufficient differences, Δ_RN_, between mean values of all parameter estimates except the number of receptors, N_ch_, which was changed from 419 ± 62 for Model R to 942 ± 228 for Model N (Figure 4E, compare red vs black boxes). Δ_RN_ for N_ch_ was 124.9% and did not fall within the range of its SE (24.2%). At the same time, Δ_RN_ for other parameters fell within the respective standard error (*SE*) range: *k*_off_: Δ_RN_ = 0.3% (*SE* = 1.7%), *a*: 1.3% (1.7%), *b*: 7.1% (18.0%), *i*_ch_: 0.8%(1.6%), P(o, peak): 8.3% (18.9%) and it was possible to conclude that the number of receptors was the only altered parameter in this case.

Thus, with ML NSFA it becomes possible to distinguish between alteration in receptor channel gating and receptor number, which nonetheless resulted in visually indistinguishable postsynaptic currents.

### Estimation of unitary current from macroscopic currents generated by receptors having different kinetic schemes

**Figure 5:**
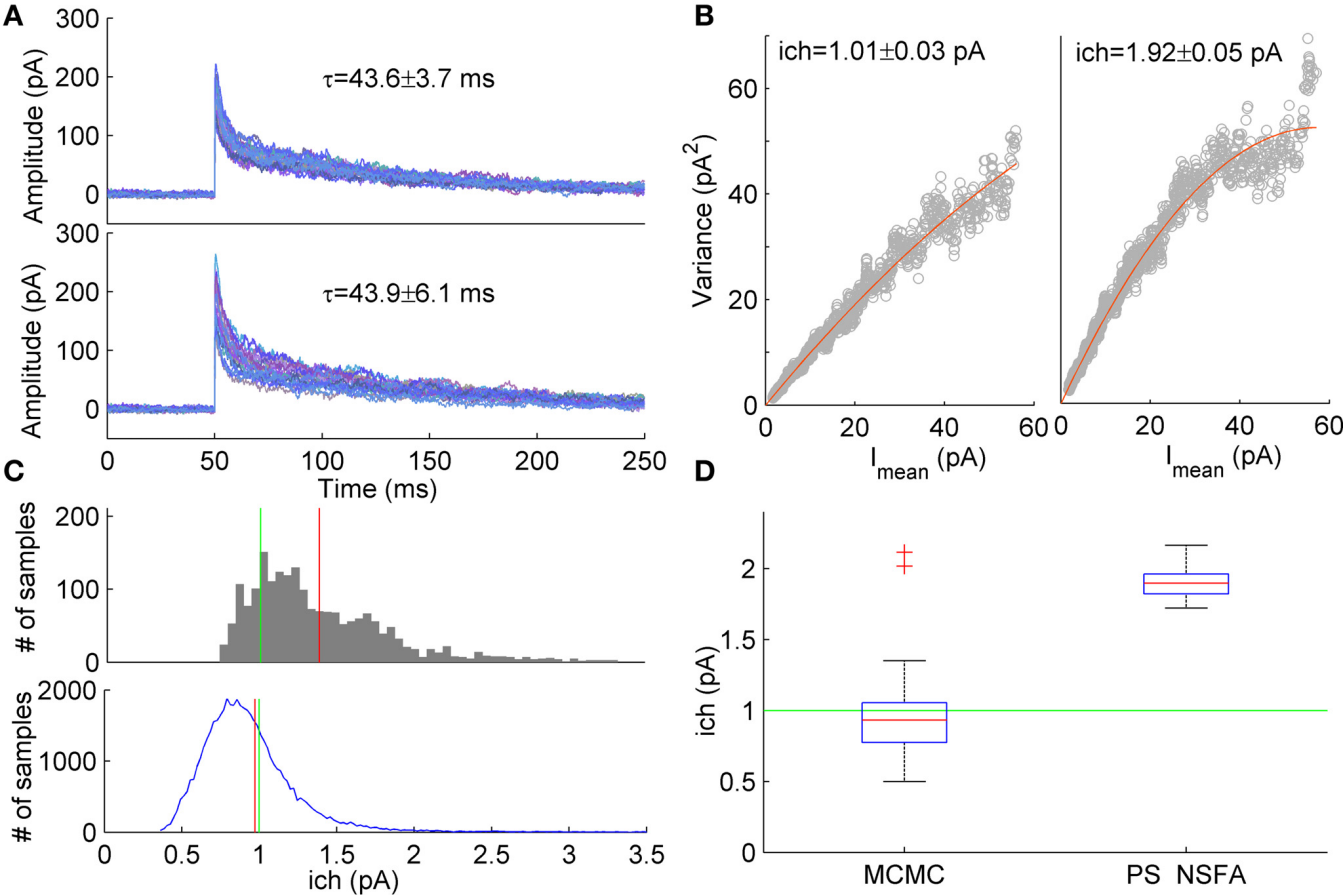
**Estimation of unitary current from macroscopic currents generated by receptors having different kinetic parameters. (A)** *Upper panel*. An example of 20 currents generated with a 7-state kinetic scheme of GABA_A_ receptor (see Figure 1A). *Lower panel*. Second group of 20 currents generated with similar model in which several parameters (*k*_off_, *d*_2_, *r*_2_) were varied randomly from current to current (uniformly in ± 20% neighborhood of their standard values, see Methods and Figure 1A). The unitary current in both groups of currents was the same, *i*_ch_ = 1 pA. Mean ± *SD* of decay time calculated over 1000 currents was 43.6 ± 3.7 ms and 43.9 ± 6.1 ms for the first and second group of currents, respectively and is shown above the traces. **(B)** Variance *vs* mean dependencies for 250 peak-scaled currents generated with (right) and without (left) variation of the channel kinetic model parameters (gray dots), and their approximation by the quadratic function (red line). **(C)** *Upper panel*. Sampling distribution of unitary current estimates obtained by MCMC sampling from the likelihood distribution of single synaptic current. *Lower panel*. Sampling distribution aggregated over 50 single current likelihood distributions. Mean of the sampling distributions and true value of unitary current are shown by red and green line, respectively. **(D)** Box plots show the statistics of the mean unitary current estimates obtained with MCMC sampling from the likelihood distributions for the group of 50 currents with varying rate constants (left) in comparison with the statistics of PS NSFA estimates obtained from the group of 250 currents with varying rate constants (right). On each box plot, the central mark is the median, the edges of the box are the 25th and 75th percentiles, the whiskers extend to the most extreme data points not considered outliers, and outliers are plotted individually by red crosses.

The key assumption of the PS NSFA is that all receptors in a particular synaptic connection under study have identical kinetic properties (Silver et al., 1996). As a result, all variance in the currents could be attributed to the stochastic nature of the channel gating rather than to the variability in their kinetics. In fact, this assumption could be violated since receptors in the synaptic connection could have different subunit composition or could be differentially modulated (Popescu and Auerbach, 2003) and a set of receptors contributing to each postsynaptic current could vary from trial to trial. In this case PS NSFA overestimates the unitary current and this overestimation could be quite significant even if the difference between receptor kinetic rates is so small that it could be hardly noticed from the observation of synaptic currents (see Figure 5A and below).

Using likelihood approximation it is possible in principle to estimate unitary current and other parameters independently for each individual synaptic current. To test this possibility we have conducted a series of computational experiments. A group of 1000 synaptic currents was simulated using 7-state kinetic scheme of GABA_A_ receptor channel (Mozrzymas et al., 2003, see scheme in Figure 1A) and the other 1000 currents were simulated using similar scheme in which several parameters (closing rate, *k*_off_, desensitization rate, *d*_2_ and resensitization rate, *r*_2_) varied between trials randomly and uniformly in the range of ±20% of parameter values that were used to generate the first group of currents. In both cases the unitary current was set at 1 pA and colored background noise (*SD* = 3 pA) was added to the simulated currents (see Section Simulation of Macroscopic Synaptic Currents in Methods for details). Figure 5A demonstrates that both groups of currents had similar waveforms and their decay times were almost identical although variability of decay times in the second group was slightly higher (Mean ± *SD*: 43.6 ± 3.7 ms vs. 43.9 ± 6.1 ms, *N* = 1000 currents). Nevertheless, variance vs. mean curves for these two groups of currents differed significantly (Figure 5B) and for the second group unitary current appeared to be 1.9-fold overestimated by PS NSFA (Mean ± *SE* was 1.01 ± 0.03 pA for the group of currents without variation of parameters vs 1.92 ± 0.05 pA for the group of currents with variation of *k*_off_, *d*_2_, and *r*_2_; *N* = 250 currents; true value was 1 pA).

To the contrary, when ML NSFA was applied to the group of currents with varying rate constants and log-likelihood of each current in the group was optimized independently, a reasonably accurate estimate of unitary current was obtained (Mean ± *SD* = 0.89 ± 0.23 pA, *N* = 50 currents). Standard error of mean unitary current estimate was very low (*SE* = 0.033 pA), but bias from the true value (1 pA) was significant. We have noticed that the cause of this bias is the skewed shape of the likelihood distribution of a single simulated synaptic current, which means that for the case of single current the maximum likelihood value of unitary current is not the most common value. An example of the typical distribution of unitary current obtained by sampling from the likelihood distribution for a single simulated macroscopic current using the slice sampling Markov chain Monte Carlo method (MCMC, 2000 samples) is shown in Figure 5C (upper panel). It can be seen that the distribution maximum significantly differs from the distribution mean (red vertical line). Therefore, in order to obtain “typical” values of unitary current, mean values of unitary current were also estimated by slice sampling from the likelihood distributions obtained for individual synaptic currents (1000 MCMC samples for each current) from the same group of 50 currents. The resulting distribution of unitary current estimates obtained by accumulation of all 50 distributions for individual currents is represented in Figure 5C (lower panel). The final estimate of unitary current was obtained by averaging over *N* = 50 mean unitary currents and was in perfect agreement with its true value (Mean ± *SD* = 0.97 ± 0.39 pA, red vertical line in Figure 5C, lower panel; *SE* = 0.056 pA). Figure 5D shows statistics of the mean unitary current estimates obtained with MCMC applied to likelihood distributions of individual currents (left box, *N* = 50) in comparison with the same statistics obtained with PS NSFA applied to individual currents as described above (right box, *N* = 250, *n* = 50 bootstraps). It is clearly seen that, contrary to MCMC, PS NSFA significantly overestimates unitary current (green line indicates true value, 1 pA). Among the other model parameters only the number of liganded channels, N_ch_, and the resensitization rate, *r*_2_, were estimated with MCMC with relatively high accuracy. The desensitization rate, *d*_2_, and GABA unbinding rate, *k*_off_, were estimated in order of magnitude. The median of the absolute difference between estimates of model parameters and their true values for *k*_off_, *d*_2_, *r*_2_, *i*_ch_, and N_ch_ was 191, 188, 22, 31, and 35% of their true values, respectively.

We conclude that the mean values for several parameters of the synaptic receptor model, such as the unitary current, the number of channels and the peak open probability, can be estimated with a reasonable accuracy using ML NSFA or MCMC sampling from the likelihood distribution of each individual current in the group of currents even if these currents were mediated by receptors having different kinetic models.

## Discussion

In this study we have further developed a new maximum likelihood method that we suggested earlier (Stepanyuk et al., 2011) and applied it to analysis of simulated macroscopic currents, in which the number of receptors exposed to a neurotransmitter varied from trial to trial. In the newly developed method, ML NSFA, the number of liganded receptors was first optimized for each macroscopic current and then these estimates were used to maximize the log-likelihood in order to obtain a set of kinetic model parameters as it was described earlier (Stepanyuk et al., 2011).

We explored the performance of ML NSFA with several different kinetic schemes of varying complexity and varying conditions relevant for real synaptic transmission. It was shown that contrary to PS NSFA (Traynelis et al., 1993) ML NSFA could estimate not only the unitary current of synaptic receptor channel but also multiple conductance levels, the number of liganded receptors, peak open probability and some kinetic constants from the experimentally realistic number of simulated postsynaptic currents. We have also evaluated the accuracy of ML NSFA compared to PS NSFA with respect to estimating the unitary current and found it 2-fold more accurate for a few (5–30) macroscopic currents. ML NSFA estimation of the unitary current was robust even when currents were generated by receptors having different kinetic parameters, the case when PS NSFA is not applicable. Thus, our results demonstrate that ML NSFA that takes into account correlations between different time points of a macroscopic currents and computationally scales linearly with the number of channel states (Stepanyuk et al., 2011) quantitatively and qualitatively outperforms currently available approaches for analysis of kinetic schemes of synaptic receptors.

## ML NSFA applicability to analysis of synaptic receptor properties

Noise analysis of macroscopic currents remains a useful tool for determining the properties of different ligand- and voltage-operated channels (Traynelis and Jaramillo, 1998). Moreover, PS NSFA, the most frequently used noise analysis approach, is the only approach that can be applied to analysis of channels with an unusually low unitary conductance (Swanson et al., 1997) and receptor channels localized at synapses (Traynelis and Jaramillo, 1998). At the same time the unitary current is virtually the only parameter that can be reliably obtained from this type of analysis (Traynelis et al., 1993; Silver et al., 1996). To the best of our knowledge, kinetic rates have never been estimated for any synaptic receptors in their intrinsic environment. Peak open probability of receptors and the number of receptors bound with a neurotransmitter could not be also directly analyzed by any current approach. Possibility to estimate the unitary current and some kinetic rates using a few simulated postsynaptic currents demonstrated in this study allows for the first time to follow a time course of receptor remodeling in one and the same synaptic connection. Having in mind that estimation of some receptor parameters with accuracy of 10% can be obtained from 10 macroscopic currents (Figures 1, 2), which can be collected in routine electrophysiological experiments for about 30 s, dynamics of receptor remodeling can be followed with a time course of several measurements per minute. It can be, for example, used for studying of modal gating, which refers to low probability rearrangements in receptor structure producing a substantial change in the overall pattern of channel opening (Popescu, 2012). Modal switches can be observed in single channel recordings of most ionotropic ligand-gated channels (Popescu, 2012) but it has never been directly demonstrated for synaptic receptors located in their intrinsic environment in a response to synaptic release of neurotransmitter. Modal gating may result not only in the different unitary conductance of receptors but also in changes in their gating and peak open probability (Popescu, 2005; Lema and Auerbach, 2006; Zhang et al., 2008; Poon et al., 2010; Prieto and Wollmuth, 2010). Moreover, in many cases, especially for the instance of NMDA receptors, substantial changes in gating, and peak open probability is observed without changes in the unitary conductance (Popescu, 2005; Zhang et al., 2008). Thus, such remodeling of synaptic receptors cannot be, in general, revealed by PS NSFA, while ML NSFA should certainly uncover it due to intrinsic ability to estimate some kinetic constants and peak open probability (Figures 1, 2). The modal gating is slow (Popescu, 2005; Zhang et al., 2008; >5 min) and agonist- and stimulus-sensitive (Armstrong and Gouaux, 2000; Poon et al., 2010). Thus, it looks potentially plausible to synchronize synaptic receptor switching between different modes for a set of synaptic receptors in a given synaptic connection and to study the modal gating of synaptic receptors in their intrinsic environment by means of ML NSFA. For example, multiple conductance levels observed in modal gating of GluA2 AMPA receptors (Prieto and Wollmuth, 2010) or different open channel probabilities found for the type 2A isoform of NMDA receptors (Popescu and Auerbach, 2003) can be resolved from the respective postsynaptic currents (Figures 2–4).

Moreover, different types of AMPA receptor regulation that occur during LTP or LTD expression, such as changes in receptor trafficking (Huganir and Nicoll, 2013), in interaction of AMPARs with auxiliary subunits (Khodosevich et al., 2014) or adapter proteins that could lead to changes in receptor kinetics (Studniarczyk et al., 2013), phosphorylation-evoked changes in unitary current and peak open probability (Traynelis and Wahl, 1997; Derkach, 2003) could be potentially resolved with ML NSFA applied to the respective postsynaptic currents. Studies of developmental, pathological, plastic, and tissue specific modifications of synaptic receptors (Kittler et al., 2004; Lüthi et al., 2004; Palmer, 2006; Stubblefield and Benke, 2010) including changes in receptor subunit composition and trafficking (Ruiz et al., 2005; Patten and Ali, 2007) that have been earlier analyzed by PS NSFA may now also obtain a second wind due to a possibility to evaluate many parameters of the respective synaptic receptors.

Conclusions about mechanisms of synaptic receptors modulation that are based solely on the analysis of the amplitude of postsynaptic currents or unitary current might be misleading. Indeed, stable unitary conductance might be accompanied by changes in receptor gating that may lead to an increase in the total charge transferred via a single synaptic receptor (Figure 4). At the level of macroscopic current it would result in an increase of current amplitude without substantial changes of its waveform (Figure 4). Together with absence of changes in the unitary conductance reported by PS NSFA it would be interpreted as presynaptic modification or an increase in the number of postsynaptic receptors. At the same time ML NSFA would certainly reveal changes in postsynaptic receptor gating.

The new approach also allows separate estimation of kinetic parameters of synaptic and extrasynaptic receptors expressed in the same neuron. For that, a set of postsynaptic currents necessary for evaluation of synaptic receptor model parameters must be initially recorded. Then strong presynaptic stimulation that can activate the whole set of synaptic terminals innervating the neuron under study should be performed in the presence of an irreversible use-dependent inhibitor of the respective synaptic receptors (e.g., picrotoxin for GABA_A_ (Olsen, 2006) or MK-801 for NMDA (McAllister and Stevens, 2000) receptors, respectively). Next, several different agonist concentrations should be sequentially applied to the preparation in order to activate the extrasynaptic receptors and to record the respective transmembrane currents. Analysis of these macroscopic currents by ML NSFA or some of previously developed approaches (Milescu et al., 2005; Moffatt, 2007; Stepanyuk et al., 2011) would give kinetic parameters of extrasynaptic receptors.

## ML NSFA applicability to analysis of synaptic receptor number and peak open probability

PS NSFA provides only an estimate of unitary current (Traynelis et al., 1993). In spite of this, estimation of N_ch_ and P(o, peak) was performed for single mossy fiber synapses of hippocampal granule cells having saturating glutamate concentration induced by synaptic vesicles release (Silver et al., 1996). In this case variance due to quantal variability is negligible and conventional NSFA can estimate these parameters. Although saturation of postsynaptic receptors is not rare in central synapses (Auger and Marty, 1997; Perrais and Ropert, 1999, 2000; Hájos et al., 2000; Nusser et al., 2001; Biró et al., 2006) estimation of N_ch_ and P(o, peak) could not be performed for the synaptic connections with multiple release sites by conventional NSFA due to trial-to-trial variability in the number of released vesicles and, as a result, in the number of receptors exposed to neurotransmitter. Moreover, in most of the central synapses neurotransmitter does not saturate postsynaptic receptors making all current methods void in determining N_ch_ and P(o, peak). On the other hand ML NSFA suggested in this study can directly evaluate the number of receptors, N_ch_, bound with neurotransmitter by the end of fast transient of neurotransmitter concentration in a synaptic cleft and P(o, peak) defined as a fraction of liganded receptors N_ch_, opened at the peak of macroscopic current (Figure 2). Moreover, N_ch_ could be separately evaluated for each postsynaptic current (Equation 18) and open probability as a function of time, which, in particular, includes P(o, peak) (Figure 2) could be obtained from estimated kinetic rate constants (Figures 1, 3, 5). Assumptions underlying ML NSFA suggest that estimations of kinetic rates as well as N_ch_ and P(o, peak) are correct if all synaptic receptors are subjected to the same and fast neurotransmitter profile or if the receptors are saturated. For some kinetic schemes (Figure 1A) saturation or the same concentration profile for all receptors are not obligatory and fast (compared to some kinetic rates) neurotransmitter profile is the only necessary assumption for ML NSFA applicability.

ML NSFA might be generally applicable to studies of synaptic and extrasynaptic NMDA receptors, glutamate receptors that directly contribute to active properties of dendrites. In the case of synaptic AMPA and NMDA receptors the ability of ML NSFA to analyze currents with variable kinetics could be important due to significant variability of glutamate transients in the excitatory synapses, low saturation levels of both receptor types (McAllister and Stevens, 2000) and complexity of their kinetic schemes (Popescu and Auerbach, 2004).

In conclusion we would like to note that more accurate estimation of unitary current compared to PS NSFA together with possibilities to distinguish multiple conductance levels and evaluate the number of liganded receptors, peak open probability and some kinetic constants position ML NSFA as a powerful tool to study synaptic receptor properties in their native environment using experimentally recorded postsynaptic macroscopic currents.

### Conflict of interest statement

The authors declare that the research was conducted in the absence of any commercial or financial relationships that could be construed as a potential conflict of interest.
